# Computer Modeling Describes Gravity-Related Adaptation in Cell Cultures

**DOI:** 10.1371/journal.pone.0008332

**Published:** 2009-12-16

**Authors:** Ludmil B. Alexandrov, Stoyana Alexandrova, Anny Usheva

**Affiliations:** 1 Harvard Medical School, Beth Israel Deaconess Medical Center, Boston, Massachusetts, United States of America; 2 Department of Applied Mathematics and Theoretical Physics, University of Cambridge, Cambridge, United Kingdom; 3 Department of Physics, Bryn Mawr College, Bryn Mawr, Pennsylvania, United States of America; University of Zurich, Switzerland

## Abstract

Questions about the changes of biological systems in response to hostile environmental factors are important but not easy to answer. Often, the traditional description with differential equations is difficult due to the overwhelming complexity of the living systems. Another way to describe complex systems is by simulating them with phenomenological models such as the well-known evolutionary agent-based model (EABM). Here we developed an EABM to simulate cell colonies as a multi-agent system that adapts to hyper-gravity in starvation conditions. In the model, the cell's heritable characteristics are generated and transferred randomly to offspring cells. After a qualitative validation of the model at normal gravity, we simulate cellular growth in hyper-gravity conditions. The obtained data are consistent with previously confirmed theoretical and experimental findings for bacterial behavior in environmental changes, including the experimental data from the microgravity Atlantis and the Hypergravity 3000 experiments. Our results demonstrate that it is possible to utilize an EABM with realistic qualitative description to examine the effects of hypergravity and starvation on complex cellular entities.

## Introduction

Artificial selections have been used often to create organisms with favorable heritable traits. Many evolutionary experiments have been applied to microorganisms such as *Escherichia coli*. Some of these experiments examine the genetic changes in multiple populations of bacteria stemming from a common ancestor that was propagated for thousands of generations [1], [2]. Others focus more specifically on the effect of starvation in stationary phase [3]. Our study utilizes an evolutionary agent based model (EABM) to simulate the effects of hypergravity on specific cellular cultures undergoing starvation. The model is validated with experimental parameters for *E. coli* bacteria. Agent based modeling has been widely used to describe economical, social, and biological systems as well to study their overall behavior [4]–[12]. EABM simulations allow capturing sophisticated behavior patterns while using simplistic calculations [7], [13]. In our model each cell is represented as an evolutionary agent, viz. in addition to all the rules guiding the agent its characteristics are stochastically heritable, thus enabling the future cells' generations to evolve during the simulation.

The results from our simulations show that the combined treatment of starvation and hypergravity may affect cellular phenotype after several generations, allowing a specific natural selection. Our results are consistent with the general conclusions from the Atlantis Space Shuttle and Hypergravity 3000 experiments [14], [15]. In addition to the utilization of our EABM to investigate the effects of hypergravity, the study provides an insight to the significance of this type of artificial selection.

### *Escherichia coli* Colony at Normal Gravity

Due to typical lack of pathogenecity, relatively small genomic size, and ease of growth, *E. coli* is a model organism studied intensively. *E. coli* is an aerobic motile bacterium, which moves with rotating flagella while consuming nutrients and dividing roughly every 20 minutes. On average, cells migrate in the environment by picking directions at random. Thus, the cellular movement could be modeled as a random walk [16]. The main bacterial characteristics that are used to describe a virtual *E. coli*, i.e. the agents in our EABM, are summarized in Table 1.

**Table 1:** Biological features of *E. coli* cells used to describe our EABM agents.

Biological Trait	Average Value	Model Analogy	Model Value Range
Average Speed	50 µm/sec	Cell Speed	1–4 links per computer step [l/cs]
Number of Cell Flagella*	5–10	Cell Flagella	5–20
Average Flagella Length*	2.5 µm	Flagella Length	5–15
Division Time	20 min	Steps For Division	72 computer steps [cs]

* Please note that the number of flagella can reach 20 due to the starvation and hypergravity conditions and that the length of flagella in the model is expressed in arbitrary and normalized units according to the formula for the average agent speed.

It is well-known that when left in a given nutrient niche, *E. coli* first consume the available local nutrients and then enter a stage of starvation. In this stage, the traits that enhance the adaptiveness of the organisms are propagated in future generations through natural selection [13]. The *rpoS* gene is switched on under starvation conditions and its genetic expression facilitates the bacterial entry into the stationary phase. This gene is crucial for bacterial survival and it is expressed to facilitate cellular accommodation to hostile environmental changes [17], [18]. Expression of the gene significantly reduces metabolic activities to preserve cellular energy. In our EABM we implemented the expression of the *rpoS* gene by modeling the agents to spend less energy and stop cellular division under starvation conditions. The *rpoS* gene expression is modeled as a heritable trait.

Recently, it was demonstrated that *E. coli* divides asymmetrically [19]. Namely, after the division each of the two daughter cells inherits a new and an old pole; the poles contain *slightly different* components. In the next division, when a daughter cell divides, one of the progeny cells inherits the old pole again and the other one a brand new pole. The latter lineage cell inherits a so called old pole and a new pole. Cells with older poles have reduced growth rate compared to cells with younger poles [19]. In our model, for simplicity, we implemented the asymmetrical divisions by random differences of the heritable characteristics taken from a Gaussian distribution.

### Cell Cultures in Different Gravity Conditions

At the Space Shuttle Atlantis Mission STS-115, evidence was found that microgravity triggers gene expression and changes cellular phenotype [14]. Statistically significant two-fold difference in the gene expression was observed between the flight-in-space and ground samples of *Salmonella typhimurium* colonies [14]. In response to microgravity conditions, *Salmonella* taken from Earth to space demonstrated increased virulence, resistance to environmental stresses (osmotic, acid, thermal), and survival in macrophages, as well as global changes in gene expression at transcriptional and translational levels [14]. Hence, transfer of cellular colonies, from Earth's gravity to the space's microgravity, alters genotypic and phenotypic responses.

Recently, human monocyte cells have been cultured in hypergravity via the Hypergravity 3000 (HG3K) machine [15]. After ten days of exposure the hypergravity treatment resulted in an overall decreased incidence of phagocytosis and vitality. It can be concluded that in general hypergravity can slow down the cellular processes. The idea is also supported by the seminal research [20] suggesting that cellular cytoskeleton responds to gravity by triggering different cellular mechanisms.

The results from our EABM simulations are consistent with the Hypergravity 3000 and Atlantis Space Shuttle experiments. Our simulations were also able to show that complex cellular entities could exhibit phenotypic changes after several generations in *hypergravity*.

## Methods

In agent-based modeling, cell cultures are simulated on a micro level by emulating the actions of autonomous decision-making entities: virtual cells (i.e. agents). Every agent makes decisions based on a predefined set of rules while having limited knowledge for the system as a whole. In our model each bacterium is represented by an agent located on a virtual Petri dish, modeled by a two-dimensional lattice with “impenetrable” boundaries. The model focuses on the following actions: agent's movement, nutrients consumption, energy exhaustion, division, gene expression, inheritance, and death. Each agent (i.e. each virtual *bacterium*) has a set of heritable characteristics which include (i) number of flagella, (ii) average flagellum length, (iii) nutrients consumption rate, (iv) rate of the *rpoS* gene expression (see below rule# 10), (v) starvation and preservation characteristics (the level at which the *rpoS* gene expression decreases energy exhaustion).

Every agent makes decisions based on the following rules:

1. An agent is always located on a site of the virtual Petri dish (i.e. a two-dimensional lattice).
2. An agent can never leave the Petri dish unless it dies.
3. An agent can only be located at one site on the Petri dish; however, there could be multiple agents on the same site. This is due to the nature of the modeled Petri dish; viz. each site represents a whole area which corresponds to the simulated agents' Brownian motion on average [16].
4. An agent dies (disappears from the virtual Petri dish) when its energy goes below a predefined energy threshold.
5. An agent consumes nutrients only when they exist at the current Petri dish site.
6. An agent has a predefined maximum of nutrients which it can consume before moving to a different site.
7. The amount of consumed nutrients is removed from the Petri dish sites.
8. An agent exhausts energy proportional to its movement, i.e. proportional to its average speed.
9. When an agent divides it exhausts some of its energy and the remaining one is asymmetrically inherited by its daughter cells.
10. An agent expresses the *rpoS* gene when there is lack of nutrients for predefined length of time (i.e. computer steps). This length of time is an inheritable characteristic.
11. When an agent expresses the *rpoS* gene it slows down its metabolic processes, viz. the agent begins to exhaust smaller than usual energy for its movement.
12. An agent can divide only if it has sufficient energy for two new daughter cells, enough time has elapsed since the last division, and the *rpoS* gene is not expressed.
13. When an agent divides, its characteristics (i.e. their average values) are inherited asymmetrically by two daughter cells. This is modeled by making the first daughter cell identical to the mother cell. The characteristics of the second daughter cell are chosen to randomly deviate from the parent's characteristics. The initial values of all heritable traits have been chosen to correspond to well-known biological characteristics.
14. At every step, the agent's speed is chosen randomly from a normal distribution with a mean value (i.e. V_AVG_) and dispersion which are calculated from its heritable characteristics.
15. Every agent chooses its direction randomly as an evenly distributed angle between 0 and 360 degrees. To find the agent's next position on the Petri dish, the crossection between the chosen random direction and a circle with radius equal to the agent's speed is used. The closest lattice site to this crossection determines the next position of the agent.
16. The average agent's speed (i.e. V_AVG_) is calculated from inherited characteristics and gravity level, viz. (Formula 1)
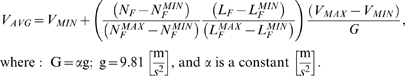
Formula 1's parameters are described in Table 2. The speed is proportional to the number of the flagella and the length of flagella. It is assumed that the average speed is inversely proportional to the Earth's acceleration *g*. This assumption is based on the fact that gravity decreases (at a first approximation) the mobility of a cell by applying pressure on its cytoskeleton [20].

**Table 2:** Average speed characteristics.

Min/Max Characteristics	Biological Meaning	EABM Value
V_MIN_	Cell Minimum Velocity	1 links per computer step [l/cs]
V_MAX_	Cell Maximum Velocity	4 links per computer step [l/cs]
N^MIN^_F_	Cell Minimum Number of Flagella	5
N^MAX^_F_	Cell Maximum Number of Flagella	20
L^MIN^_F_	Cell Minimum Flagella Length	5
L^MAX^_F_	Cell Maximum Flagella Length	15

Following the above rules, a virtual cellular colony with *Escherichia coli* parameters was simulated to develop and grow at every computer step, and thus validate the model. At the beginning of each simulation, nutrients are distributed on the Petri dish, i.e. on the two-dimensional lattice. Every run, eighty-five percent of the sites are randomly chosen to contain nutrients. The nutrient quantity at each of the sites is obtained randomly from a Gaussian distribution with the same average value and standard deviation for every run.

At the beginning of every simulation, when there is abundance of nutrients, the cell's generations are synchronous, i.e. every bacterium has enough energy to divide every 20 minutes. However, as the simulation progresses the resources become scares and as a result the bacteria become asynchronous since there are not enough nutrients for every cell to divide at the same time.

The results were averaged over a set of 100 simulations for each of the different values of the gravity (i.e. *g* = 1 and *g* = 1.5). Every simulation lasted for 26,000 computer steps which correspond to ∼120 hours of *E. coli* life, at which a steady plateau of the bacterial growth is reached. The random seeds we used were different for each simulation in a given set; however, identical random seeds were used for the two sets of simulations modeling different gravity conditions. Every run starts with an initial population of 1000 *E. coli* cells randomly spread in a region of the virtual Petri dish. The cell's heritable characteristics are generated from Gaussian distributions with biologically meaningful average values and narrow standard deviations. During every simulation the system contained ∼10^8^ living agents (i.e. *Escherichia coli*) at the most abundant point. Throughout each run, we monitored the evolution of the: (i) average cell's growth rate, (ii) average length and number of flagella, (iii) average time interval for *rpoS* gene expression, (iv) average bacteria speed, and (v) average nutrients quantity on the lattice.

We scaled the initial energy and nutrients to qualitatively match the experimental results of *Escherichia Coli* growth in conditions of normal gravity and starvation. Namely, the parameters of the model such as: rate at which an agent consumes nutrients, value of the *E. coli* initial energy, quantity of the nutrients distributed on the lattice, number of steps needed for *E. coli* to enter preservation state (i.e. to express the *rpoS* gene), and values of the V*_MIN_* and V*_MAX_* were scaled to reproduce qualitatively the experimental data for *E. coli* behavior in starvation [18], [21]. Therefore, we scaled the parameters of our model to match a validated bacterial growth behavior in normal gravity conditions and starvation [22], [23].

All computer simulations were performed on the Orchestra Linux cluster at Harvard Medical School.

## Results

### Model Validation

**Figure 1:**
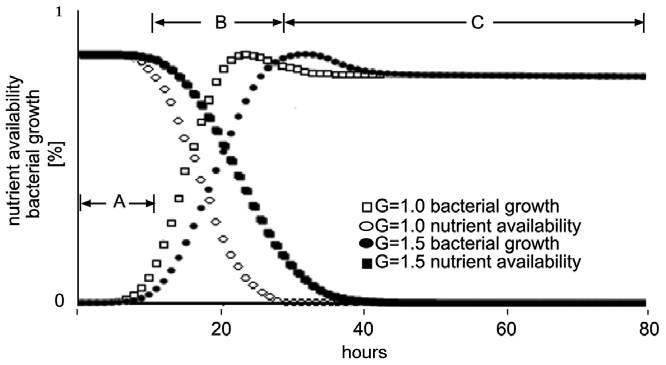
Average bacterial growth rate and nutrient availability at different gravity levels. The bacterial growth curves are shown with squares – at normal gravity, G = 1.0, empty squares; at hypergravity G = 1.5, full squares. Time dependent changes in nutrients amount on the lattice due to bacterial consumption is shown with empty circles for G = 1.0 and full circles for G = 1.5. Bacterial growth and nutrients availability is shown on the Y-axis. Maximum bacterial growth corresponds to ∼10^8^ cells and 0 corresponds to 1000 cells. Availability of nutrients on 85% of the lattice corresponds to the maximum nutrients availability. The X-axis depicts the time, rescaled from computer steps to hours. Domain A corresponds to the *lag* phase of the bacterial growth; domain B - *logarithmic* growth phase; domain C - long-term *stationary* phase.

Our primary goal was to utilize the EABM, viz. to reproduce cellular growth and cellular responses (due to natural selection) to environmental factors such as starvation, as well as to create a qualitative prediction of the bacteria behavior at different gravity conditions. The model was validated via comparison with experimental data for *E. coli* growth at normal gravity and starvation [18], [21], [23]. The bacteria growth presented on Figure 1 demonstrates qualitative consistency between the simulation data and the experimental results [20], [23]. The *lag* phase as well as the *logarithmic* and the *long-term-stationary* phase can be clearly seen at the growth and the nutrients consumption curves for both gravities. The hump on each of the growth graphs (domain B) corresponds to the position where cells begin to enter the state of preservation due to initiation of the *rpoS* gene expression [19].

The simulation data demonstrate the expected growth rate at normal gravity. At hypergravity, the model predicts a decrease in cellular growth as seen on Figure 1. Additionally, decrease in mobility as well as differences in the length and number of flagella was observed. Please note that the standard deviation is not shown on the figures since it is negligible.

### Gravity-Related Changes in Bacteria Behavior

**Figure 2:**
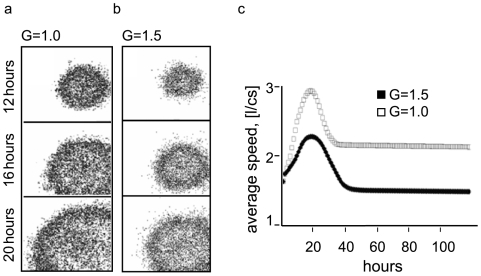
Culture growth and colony formation in conditions of normal gravity and hypergravity. Identical set of initial random seeds are used for the simulations at normal gravity and hypergravity. (a) Predicted character of movement in normal gravity (G = 1.0) at different time points as shown on the left. The dots correspond to individual cells. (b) Predicted character of movement in hypergravity (G = 1.5) at the different time points. The walls of the virtual Petri dish are shown on the X and the Y. (c) Predicted average cell speed in normal gravity (an empty square) and hypergravity (a full square). X-axis depicts the time rescaled from computer steps to hours. Y-axis shows the average cell speed in links per computer step (hours).

Depending on food availability and the environmental conditions, bacteria grow in colonies on the surface of the agar. It is well known that the colony formation can have various types of collective motion. The state of bacterial growth of an average colony at various time intervals and at different gravity conditions is shown on Figure 2. Every panel presents a colony after 10 hours development. Our simulations demonstrate diffusion-like (i.e. Brownian [16]) character of the bacterial motion. The colony spreads out and populates the virtual Petri dish. The bacteria exhaust the nutrients first at the center of the colony where the bacterial density is higher. Next, the colony spreads onto sites with higher nutrient availability. The colony in hypergravity (Figure 2, panel b) spreads with a slower rate and exhibits a lower density of cells but similarly mimics a diffusing wave moving outward, i.e. towards the more nutrient-rich environment.

Data presented on panel c support a conclusion that at normal gravity the bacteria are faster in reaching the nutrient-rich areas than bacteria at higher gravity. However, the number of the fast migrating bacteria declines once the nutrient levels decrease in both gravities (panel c). The slow moving bacteria display enhanced adaptiveness due to reduced energy expenditure. When the nutrients are exhausted, different constant average speeds are established for both gravities.

### Gravity-Related Changes in *rpoS* Gene Expression

**Figure 3:**
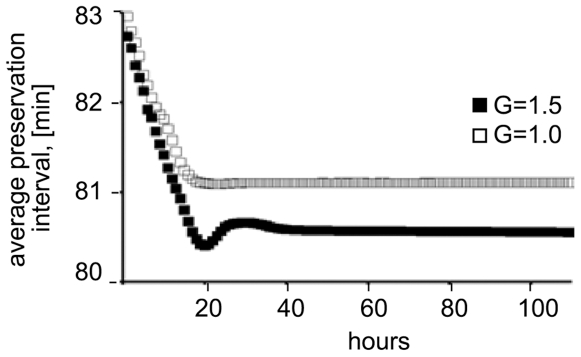
Average time required for initiation of the *rpoS* gene expression. The predicted average time for initiation of *rpoS* gene expression in hypergravity conditions is shown with full squares; normal gravity - empty squares. The X-axis depicts the time, rescaled from computer steps to hours; Y axis - average interval after which the preservation *rpoS* gene is expressed.

In our EABM we postulated that the gravity slows down the motion proportionally to the applied acceleration (Formula 1). Every individual cell in the hypergravity moves slower and therefore has difficulties finding nutrients. This makes favorable an earlier expression of the *rpoS* gene, which leads to preservation of the cellular energy. Hence, after several generations in hypergravity, the average cell begins to express the *rpoS* gene earlier than the average cell at normal gravity conditions (Figure 3).

### Gravity-Related “Enhanced” Cellular Adaptiveness

**Figure 4:**
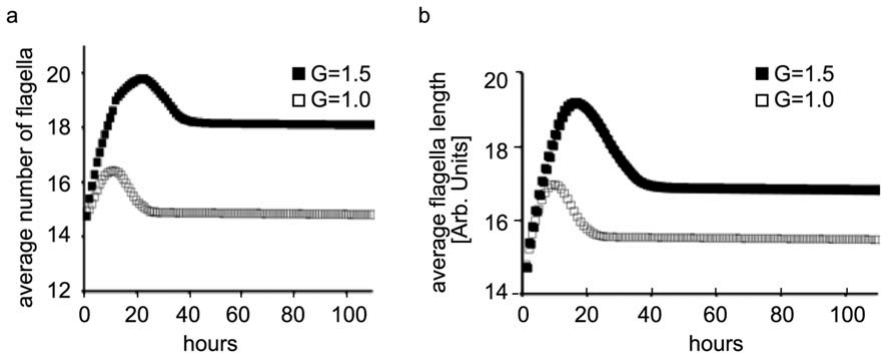
Changes of flagella under normal and hypergravity conditions. (a) The average number of flagella is plotted on the Y-axis. The X-axis depicts the time, rescaled from computer steps to hours. (b) Predicted time dependent change of the average length of flagella in the cell culture. The Y-axis shows the average flagella length in arbitrary units. The X-axis depicts the time rescaled from computer steps to hours. Empty squares correspond to normal gravity conditions; full squares correspond to hypergravity conditions.

In our model the average cellular speed is proportional to the number and length of flagella (Formula 1). The simulations show that the average number (Figure 4, panel a) and length of flagella (panel b) initially increases. This is natural since the cells with an enhanced motility will have better chance to find nutrients and therefore survive. Mostly such cells survive to pass their characteristics to the next generations. This process continues as long as the available nutrients are sufficient. In our model, the average speed increases to an optimal value dictated by a natural “penalty.” Namely, the exhausted energy per jump is proportional to the jump's length; therefore the exhausted energy is proportional to the agent's motility. This “penalty” limits the average bacterial speed. Under hypergravity conditions the only difference is that the average speed as well as the number and length of flagella are different from those under normal gravity conditions (Figure 4). After the nutrients have been exhausted, another trait, viz. the trait for early energy preservation, becomes more beneficial for the bacterium survival. Hence, the bacteria with this trait naturally dominate the culture after several generations. The colony begins to accumulate cells with a decreased average speed, i.e. cells with smaller number (panel a) and shorter length of flagella (panel b).

These results, combined with the difference in average speed of migration (Figure 2, panel c), indicate that the final cell generation grown in hypergravity has a lower average speed, which is consistent with the Hypergravity 3000 machine experiment [15]. Interestingly, the simulations show that at the hypergravity stationary phase, the bacteria have higher number and length of flagella but with average speed less than the one at normal gravity conditions. Evidently, the hypergravity conditions make favorable more and longer flagella in order for the average cell to reach the optimum speed. This is a central point of our work. According to the simulations, an average bacterium at hypergravity conditions has *lower average speed* but *more and longer flagella*. If such cells are placed at lower gravity, the greater number and longer length of the flagella will lead to higher average speed in comparison to the average speed of cells grown at normal gravity conditions. Hence, a cell generation grown in hypergravity conditions will be faster and functionally more efficient than the cells raised in normal gravity. Equivalently a cell culture raised in normal gravity and transferred to micro-gravity conditions would be faster and, surprisingly, highly functional.

## Discussion

The presented here EABM describes evolutionary adaptation of cellular colony grown for generations in simulated starvation and hypergravity. In hypergravity, the adapted bacteria have acquired longer and greater number of flagella as well as the function to express the *rpoS* gene earlier. These new characteristics are a direct result from the natural selection in hostile environment and act as a compensation for the decreased cell motility. The generated characteristics increased the overall adaptiveness of the average cell. With such traits, a hypergravity-raised bacterial colony would have an advantage if placed back in lower gravity conditions, which we named *the gravity effect*. Hypergravity-raised cells will be faster, stronger, and will require fewer nutrients to survive.

In 2007, *Salmonella typhimurium*, a cell culture originating from Earth was placed in microgravity on the space shuttle Atlantis [14]. We are able to discuss the results from this experiment due to the fact that all important agent characteristics used in our model are almost identical for *Salmonella typhimurium* and *Escherichia coli*. In general, these two types of bacteria are very similar. This accounts for identical traits, e.g. they are both Gram-negative and have lipopolysaccharide outer membranes [24]. Both types of bacteria divide by producing two daughter cells as typical bacterial entities. Importantly, the *Salmonella typhimurium* are rod-shaped motile bacteria that possess flagella for motility [25] which is a significant characteristic of our agents. Furthermore, it was confirmed that *Salmonella typhimurium* also exhibits starvation-stress response connected with the *rpoS* gene [26], [27] which is another essential characteristic in our simulations. These similarities in division, movement, and gene expression make our model applicable for both types of bacteria. Hence, the conclusions from our simulations can be, at least qualitatively, applied to *Salmonella typhimurium*.

The Atlantis Space Shuttle experiment could be examined from the following perspective: a bacterial culture originating from Earth, at normal gravity (i.e. 1.0 g), represents a colony *grown in hypergravity* compared to the Atlantis Space Shuttle *microgravity* conditions. Accordingly, the Atlantis samples (i.e. bacteria grown in hypergravity) exhibited enhanced virulence and extracellular matrix accumulation. We believe that the Atlantis experiment provides a direct evidence for the existence of the demonstrated, by our model, *gravity effect* and is an example for hypergravity-generated cells with enhanced capabilities.

Additionally, data for the effect of hypergravity on cellular behavior come from the Hypergravity 3000 machine experiment [15]. In this experiment human monocyte cultures were placed in real hypergravity conditions. The results showed that the monocytes respond with an overall decreased incidence of phagocytosis and motility. Human monocytes are completely different from E. coli bacteria. However, we emphasize that a decreased motility, and hence reduced phagocytosis, is a natural phenomenon expected in hypergravity regardless the type of microorganism. This is consistent with the predicted by our model reduced cellular functioning under hypergravity.

It is likely that hypergravity has the potential to change cellular behavior in different ways depending on the cellular type and on the strength of the hypergravity. Importantly, this idea is supported by the seminal research [20] demonstrating that the cellular cytoskeleton responds to gravity by triggering different cell type dependent mechanisms.

In summary, we applied an evolutionary agent-based model to simulate natural selection and adaptation in bacteria in response to environmental changes. We demonstrated that it is possible to create a combination of probabilistic, adaptive, and deterministic rules that allow a realistic qualitative description of the evolution of complex cellular entities in hypergravity. Through set of rules, interactions, and simulated mechanism of asymmetrical heredity, a phenomenological description of the evolution of the agents' characteristics was defined and maintained throughout the simulations. The possibility for appearance of unfavorably mutated agents was systematically reduced by including restrictions in the simulations that are based on the environmental conditions and the agent rules. The features of the presented model are the quantitatively realistic definitions of cellular colony, cellular parameters, and the consistency between the simulations and experimentally collected data. An important future question is the simulation of adaptation of mammalian cells under high pressure.
